# Relationship between Serum Asymmetric Dimethylarginine Level and Microvascular Complications in Diabetes Mellitus: A Meta-Analysis

**DOI:** 10.1155/2019/2941861

**Published:** 2019-02-25

**Authors:** Jing Liu, Caiying Li, Wen Chen, Kuanrong He, Huijuan Ma, Boqing Ma, Pei Zhao, Lu Tian

**Affiliations:** ^1^Department of Endocrinology, Hebei General Hospital, Shijiazhuang 050017, Hebei, China; ^2^Department of Anus and Intestines Surgery, No. 1 People's Hospital in Shijiazhuang, Shijiazhuang 050000, Hebei, China; ^3^Graduate School, Hebei Medical University, Shijiazhuang 050000, Hebei, China; ^4^Department of Internal Medicine, Hebei Medical University, Shijiazhuang 050017, Hebei, China; ^5^Clinical Laboratory, Hebei General Hospital, Shijiazhuang 050017, Hebei, China; ^6^Obstetrics Department, Shijiazhuang Obstetrics and Gynecology Hospital, Shijiazhuang 050000, Hebei, China

## Abstract

**Objective:**

The purpose of the meta-analysis was to evaluate the relationship between serum asymmetric dimethylarginine (ADMA) level and microvascular complications in diabetes mellitus (DM) including diabetic retinopathy (DR), diabetic neuropathy (DN), and diabetic nephropathy.

**Methods:**

Studies were comprehensively identified by searching Web of Science, Embase, and PubMed databases up to August 30, 2018. The meta-analysis was carried out to compare the difference of serum ADMA concentrations of DR, DN, and diabetic nephropathy patients with healthy controls. The Newcastle-Ottawa Scale and the Agency for Healthcare Research and Quality were applied to assess the methodological quality. Chi-squared Q test and I^2^ statistics were applied to evaluate statistical heterogeneity. Subgroup analyses were conducted and publication bias was assessed by Egger's test.

**Result:**

Ten studies were finally entered in the meta-analysis. Statistically significant heterogeneity was observed across these studies (*I*^2^ = 77.0%, p < 0.001). Compared with DM without microvascular complications, circulating level of ADMA was significantly higher in DM with microvascular complications (all p < 0.05). Sensitivity analysis suggested that the results of this meta-analysis were shown to be stable. There was no significant publication bias (P=0.823).

**Conclusion:**

Elevated ADMA levels correlate with diabetic microangiopathies such as DR and diabetic nephropathy. ADMA may play an important role in the pathobiology of microvascular complications of diabetes.

## 1. Introduction

Diabetes mellitus (DM) is caused by a defect in insulin secretion and/or insulin resistance leading to persistent hyperglycemia, affecting millions of people worldwide [1]. Most long-term DM patients develop microvascular complications such as nephropathy, retinopathy, and neuropathy, which create substantial disability and high mortality rates in diabetic patients [2]. Therefore, it is imperative to understand the underlying mechanisms of microvascular complications in order to reduce the burden.

Studies demonstrate that endothelial dysfunction plays a vital role in the development of diabetes-associated microvascular complications [3, 4]. Asymmetric dimethylarginine (ADMA), which is an endogenous inhibitor of endothelial nitric oxide (NO) synthase, is increased in patients with endothelial dysfunction [5, 6]. Evidence demonstrates that plasma ADMA level is elevated in patients with diabetic microangiopathy such as diabetic retinopathy (DR) [7], diabetic nephropathy, and diabetic neuropathy (DN) [8, 9]. Microalbuminuria (MIC) is considered incipient diabetic nephropathy and macroalbuminuria (MAC) is considered established diabetic nephropathy [10]. However, Yasar et al. indicated that ADMA did not have any significant role in DN [11]. Additionally, Yonem et al. reported that the plasma ADMA was similar in patients without DR and patients with DR [12]. Therefore, the available evidence regarding the relationship between ADMA and diabetic microvascular complications is not conclusive.

Therefore, meta-analysis of all relevant studies was conducted to assess the association between ADMA level and diabetic microvascular complications.

## 2. Methods and Materials

### 2.1. Data Source and Search Strategy

The meta-analysis was performed based on the Meta-Analysis of Observational Studies in Epidemiology: A Proposal for Reporting (MOOSE). We comprehensively searched Web of Science, PubMed, and Embase to identify related articles published before August 30, 2018. No language restriction was imposed. Literature search was performed using keywords as follows: “N,N-dimethylarginine”; “N(G1), N(G1)-dimethylarginine”; “N(G)-dimethylarginine”; “asymmetric dimethylarginine”; “guanidine-N,N-dimethylarginine”; “N(G), N(G)-dimethylarginine” or “dimethyl-L-arginine” and “diabetic retinopathy” or “retinopathies, diabetic” or “diabetic retinopathies”; “retinopathy, diabetic” and “diabetic neuropathies” or “diabetic neuropathy” or “diabetic autonomic neuropathy” or “diabetic autonomic neuropathies” or “diabetic neuralgia”; “diabetic neuralgias” or “painful diabetic neuropathies” or “painful diabetic neuropathy” or “asymmetric diabetic proximal motor neuropathy” or “diabetic asymmetric polyneuropathy” or “diabetic asymmetric polyneuropathies” or “diabetic mononeuropathy” or “diabetic mononeuropathies” or “diabetic mononeuropathy”; “diabetic mononeuropathy simplices” or “diabetic amyotrophy” or “diabetic amyotrophies” or “diabetic polyneuropathy” or “diabetic polyneuropathies” and “diabetic nephropathy” or “nephropathy, diabetic”, “DN”. The reference lists were also scanned to acquire the additional relevant articles. We considered all potentially eligible publications regardless of language or the primary outcome to avoid biases. Additionally, a manual search from the reference list of key articles published in English was used to identify additional papers. No language or date limitations were set.

### 2.2. Inclusion Criteria and Exclusion Criteria

Study design had to meet the following criteria: the study should report the correlation of serum ADMA level in DM to retinopathy, neuropathy, or nephropathy; they were observational studies (cross-sectional, case-control study, and cohort study research); study data should be represented by mean ± SD or can be converted based on the information in the literature; studies are with control group of DM without retinopathy, neuropathy, or nephropathy; patients are of any gender, age, region, or race with DM.

We excluded studies if the same studies came from different database; if they were case reports, animal studies, and summaries of personal experience; if there were no controls or absence of DM with retinopathy, neuropathy, or nephropathy.

### 2.3. Data Extraction and Quality Assessment

The literature search, document selection, and data extraction were performed independently by two investigators (Jing-liu and Kuanrong He). The Newcastle-Ottawa Scale (NOS), a representative tool, was used to measure the quality of case-control or cohort studies [13], including three dimensions: low quality = 0-3; moderate quality = 4-6; high quality =7-9. A checklist adapted and used by the Agency for Healthcare Research and Quality (AHRQ) was used to assess quality of cross-sectional studies. A total of 11 items were involved. An item that would be scored “0” indicates a “NO” or “UNCLEAR” response; “1” indicates a “YES” response. A score of 0-3 was considered as low quality, 4-7 moderate, and 8-11 high quality.

The following data were collected: (1) first author's name; (2) publication date; (3) country of the study; (4) disease duration; (5) number of cases and controls considered; (6) mean age; (7) mean serum ADMA levels of cases and controls (presented as mean + SD). Disputes were resolved via consultation or discussion with a third reviewer (Wen Chen).

### 2.4. Statistical Analysis

We used the statistical software program Stata12.0. The data was presented as mean ± SD. Chi-squared Q test and I^2^ statistics were applied to evaluate statistical heterogeneity of changes in ADMA levels in DM with retinopathy, neuropathy, nephropathy, and controls. If there was a statistical homogeneity between studies (p < 0.05 or I^2^ > 50%), we used a random-effect model; otherwise we used a fixed-effect model [14]. Subgroup analyses were conducted by subtype of microvascular complications in type 2 diabetes (T2DM); participant (T2DM, T1DM&T2DM); different ethnicities of DM patients; and study design (case-control study and cross-section study).

Egger's test [15] was used for assessing publication bias and significant publication bias was defined as a p value < 0.05; sensitivity analysis was applied to assess the stability of the results.

## 3. Results

### 3.1. Search Results

**Figure 1:**
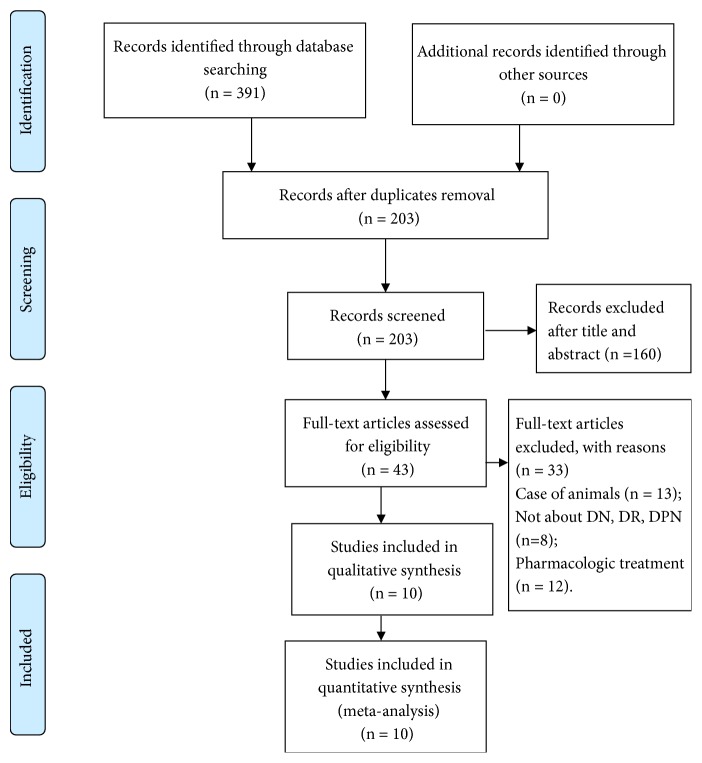
Flow diagram depicting the studies identified and included into the meta-analysis.

A flow chart of the article selection process is presented in Figure 1. A total of 10 works [7, 11, 12, 16–22] that matched the research criteria of the study design were adopted. Among them, five were case-control studies [7, 11, 16, 17, 21] and five were cross-sectional studies [12, 18–20, 22], including 942 DM with retinopathy, neuropathy, and nephropathy and 873 controls. The basic characteristics of the studies involved are displayed in Table 1. Of these 10 articles, five had two case groups (MIC group and MAC group) [16–18, 20, 22]. The 10 articles were shown to be of moderate or high quality by NOS (Supplementary Tables 1 and 2).

### 3.2. Main Analysis

**Figure 2:**
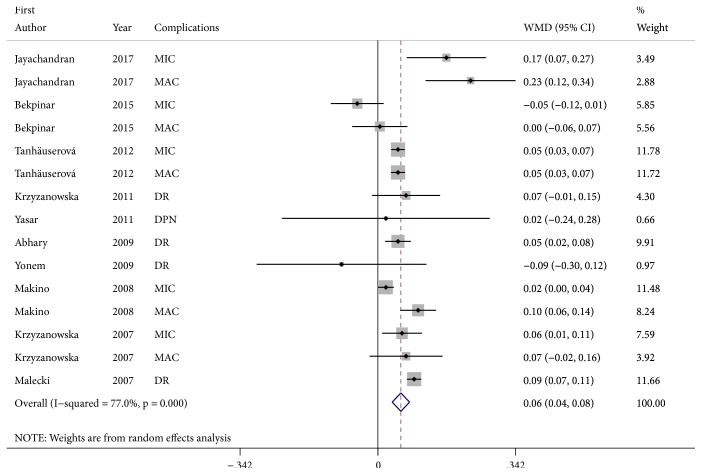
Forest plot detailing the relationship between DM with microvascular complications and serum levels of ADMA.

There was significant heterogeneity (*I*^2^ = 77.0%, p < 0.001). A random-effects model was applied. Compared to controls, DM with microvascular complications had significantly higher ADMA serum levels (WMD, 0.06; 95% CI, 0.04-0.08; p < 0.001) (Figure 2). Subgroup analysis revealed that, except DN, patients with diabetes-associated microvascular complications had higher significantly ADMA serum levels (Table 2). In addition, increased ADMA levels were found by HPLC detection (Table 2).

In the sensitivity analysis, no study individually altered the corresponding pooled WMDs (WMD&95CI: 0.046 (0.037, 0.055)–0.062 (0.053, 0.070), P<0.05), suggesting that the results of this meta-analysis were stable. Moreover, no significant publication bias was found by the Egger's test (P=0.823).

## 4. Discussion

Our evidence suggests that DM with microvascular complications had increased serum levels of ADMA.

DR, a major microvascular complication of DM, is a leading cause of preventable blindness in working-age adults. ADMA, a strong and independent predictor of endothelial dysfunction, is increasingly a focus of interest in DR. Evidence indicates that plasma ADMA levels are markedly elevated in diabetic patients with advanced retinopathy [7, 21]. This is in line with the results of our study. Subgroup analysis supports the notion that higher ADMA contributes to the pathogenesis of retinopathy. Moreover, evidence showed that higher ADMA existing in aqueous humor is associated with severe retinopathy [23]. Reducing ADMA may be used to inhibit the development of DR; further studies are needed.

Diabetic nephropathy, characterized by a decline in glomerular filtration rate and proteinuria, has become the leading cause of end-stage renal disease and the strongest predictor of mortality in diabetes [24]. ADMA is an important stimulator for oxidative stress [25], which plays vital roles in the initiation and development of diabetic nephropathy [26]. Circulating ADMA levels are increased in T2DM with nephropathy in study. Proteinuria is a traditional marker of progression of renal injury in diabetes. Experimental and clinical studies demonstrate that elevated ADMA is associated with severe proteinuria [27, 28]. Here, we show that ADMA is significantly elevated in DM patients with albuminuria.

DN is one of the major diabetic microvascular complications and mainly affects both autonomic and peripheral nerves. There is emerging evidence that ADMA is associated with DN [7]. Oxidative stress has been demonstrated to be an essential factor in the pathogenesis of DN [29], suggesting that ADMA maybe plays a pivotal role in the development of DN [25]. In this study, subgroup analysis indicated that there was no significant increase in ADMA levels in patients with DN. Nevertheless, further studies are needed to determine the association between ADMA level and diabetic neuropathy.

To our knowledge, this meta-analysis is the first that assessed the relationship between ADMA level and DM microvascular complications. However, this meta-analysis has several limitations. First, high heterogeneity was observed. A random-effects model and sensitivity analysis were used to adjust for heterogeneity. Egger's tests illustrate that no publication bias was found in any analysis. Second, subgroup analysis was not successful in detecting the source of heterogeneity. In some studies, there was a significant difference in baseline information (age, duration, biochemical indicators, etc.) between the case group and the control group. The rigor of the experimental design and the pertinence of the inclusion criteria for case groups or control groups between the various included studies may have a certain impact on the results. In addition, to a certain extent, other factors such as the type of study object and laboratory assay for serum ADMA level affected the evaluation. Moreover, the included studies are not randomized controlled trials and are thus vulnerable to potential bias.

In conclusion, elevated ADMA are associated with diabetic microangiopathies such as retinopathy and nephropathy. ADMA plays an important role in the pathobiology of microvascular complications of diabetes. We suspect that ADMA may be a target for diagnosis and treatment of microvascular manifestations in diabetes.

## Figures and Tables

**Table 1:** Characteristics of the studies included in the meta-analysis.

First Author, Year, Country	Study design	Measurement of ADMA	Paticipants	Group	Diabetes duration (years)	Criteria	N	Age, years	M/F	Disease duration (years)	ADMA, mean±SD
Jayachandran, 2017, India	case- control	ELISA	Type 2 DM	NB	NR	<30 *μ*g/mg UACR	45	46.4±8.4	28/17	NR	0.62±0.27
				MIC	NR	30-299 *μ*g/mg UACR	45	52.2±5.9	36/9	NR	0.79±0.20
				MAC	NR	≥ 300 *μ*g/mg UACR	45	51.8±6.7	34/11	NR	0.85±0.27

Bekpinar, 2015, Turkey	case- control	HPLC	Type 2 DM	NB	9.3±1.2	<30 mg/day UAER	30	54.2±1.3	12/18	9.3±1.2	0.775±0.115
				MIC	12.7±1.5	30-300 mg/day UAER	30	59.4±1.5	17/13	12.7±1.5	0.724±0.142
				MAC	14.1±1.3	> 300 mg/day UAER	30	61.8±1.36	16/14	14.1±1.3	0.78±0.153

Tanhauserováa, 2012, Czech Republic	cross- sectional	HPLC	T1DM & T2DM	NB	16(11-23)	<30 mg/day UAER	71	52(36-64)	NR	16(11-23)	0.36±0.059
				MIC	11(7-19)	30-300 mg/day UAER	115	66(56-75)	NR	11(7-19)	0.41±0.052
				MAC	17(10-22)	>300 mg/day UAER	155	65(60-74)	NR	17(10-22)	0.41±0.067

Krzyzanowska, 2011, Austria	cross- sectional	HPLC	Type 2 DM	Non-DR	9 (4–16)	ETDRS	60	62(54-69)	41/19	9 (4–16)	0.55±0.14
				DR	14 (10–17)	ETDRS	67	64 (56-73)	37/30	14 (10–17)	0.62±0.32

Yasar, 2011, Turkey	case- control	IMA	T1DM & T2DM	Non-DN	9.1±6.3	NSST	28	58.1±15.5	NR	9.1±6.3	0.51±0.51
				DN	12.2±7.4	NSST	30	62.9±11.1	NR	12.2±7.4	0.53±0.49

Abhary, 2009, Australia	case- control	HPLC	T1DM & T2DM	Non-DR	NR	ETDRS	330	NR	NR	NR	0.67±0.14
				DR	NR	ETDRS	175	NR	NR	NR	0.72±0.19

Yonem, 2009, Turkey	cross- sectional	ELISA	Type 2 DM	Non-DR	6.4±5.5	NR	41	52.6±9.4	23/18	6.4±5.5	0.93±0.42
				DR	13.5±8.3	NR	38	58.3±9.7	20/18	13.5±8.3	0.84±0.34

Makino, 2008, Japan	cross- sectional	HPLC	Type 2 DM	NB	12±8	<30 mg/day UAER	121	62±9	76/45	12±8	0.45±0.06
				MIC	14±8	30-300 mg/day UAER	71	65±8	34/37	14±8	0.47±0.07
				MAC	18±8	>300 mg/day UAER	25	66±7	12/13	18±8	0.55±0.11

Krzyzanowska, 2007, Austria	cross- sectional	HPLC	Type 2 DM	NB	9(4-14)	<30 mg/day UAER	36	62(56-69)	18/18	9(4-14)	0.55±0.11
				MIC	10(3-18)	30-300 mg/day UAER	40	63(56-72)	28/12	10(3-18)	0.61±0.11
				MAC	14(6-22)	>300 mg/day UAER	27	66(61-74)	17/10	14(6-22)	0.62±0.22

Malecki, 2007, Poland	case- control	HPLC	Type 2 DM	Non-DR	NR	NR	111	56.2±6.5	NR	NR	0.51±0.06
				DR	NR	NR	71	56.2±6.5	NR	NR	0.60±0.06

M, male; F, male; NB, normoalbuminuria; ADMA, asymmetric dimethylarginine; ELISA, enzyme linked immunosorbent assay; MIC, microalbuminuric; MAC, macroalbuminuric; HPLC, high performance liquid chromatography; UACR, urinary albumin creatinine ratio; UAER, urinary albumin excretion rate; ETDRS, the Early Treatment of Diabetic Retinopathy Study classification; NR, not reported; IMA, Immunosorbent Assay; NSST, Neuropathy Symptom Score test; DN: diabetic neuropathy; DR: diabetic retinopathy.

**Table 2:** Outcomes ofsubgroup analyses.

Outcomes	No. of studies	MD (95%CI)	*P*_A_	Heterogeneity test	
				*P*	I^2^ (%)
*All studies*	15	0.06 (0.04, 0.08)	<0.001	<0.001	77.0
*Complications*					
MIC	5	0.04 (0.00, 0.07)	0.028	<0.001	80.1
MAC	5	0.08 (0.03, 0.13)	0.002	0.003	74.6
DR	4	0.07 (0.03, 0.10)	<0.001	0.068	58.0
DN	1	0.02 (-0.24, 0.28)	0.879	-* *-	-* *-
*Area*					
Asian	4	0.12 (0.03, 0.20)	0.006	<0.001	89.6
Western	11	0.05 (0.03, 0.07)	<0.001	0.001	66.0
*Type of research*					
Case-control	7	0.07 (0.02, 0.12)	0.009	<0.001	82.6
Cross-sectional	8	0.05 (0.03, 0.07)	<0.001	0.031	54.6
*Participants*					
T2DM	11	0.03 (0.03, 0.10)	0.001	<0.001	83.2
T1DM&T2DM	4	0.05 (0.04, 0.06)	<0.001	0.997	0.0
*Measurement of ADMA*					
ELISA	3	0.12 (-0.04, 0.27)	0.148	0.007	80.0
HPLC	11	0.05 (0.03, 0.07)	<0.001	<0.001	77.3
IMA	1	0.02 (-0.24, 0.28)	0.879	-* *-	-* *-

*P*_*A*_: *P* value for test of the association; MIC, microalbuminuria; MAC, macroalbuminuria; IMA, Immunosorbent Assay; ELISA, enzyme linked immunosorbent assay; HPLC, high performance liquid chromatography; DN: diabetic neuropathy; DR: diabetic retinopathy; ADMA, asymmetric dimethylarginine.

## Data Availability

All data generated or analysed during this study are included in this published article.

## Supplementary Materials

Supplementary Materials Supplementary Table 1. Quality assessment of the cross-sectional studies. Supplementary Table 2. Quality assessment of the case-control studies.
