# Supplementary material for: Relationship between Serum Asymmetric Dimethylarginine Level and Microvascular Complications in Diabetes Mellitus: A Meta-Analysis
Source: Biomed Res Int. 2019 Feb 25;2019:2941861. doi: 10.1155/2019/2941861 (PMC6413490; doi:10.1155/2019/2941861)
Supplement: Supplementary Materials — Supplementary Table 1. Quality assessment of the cross-sectional studies. Supplementary Table 2. Quality assessment of the case-control studies. [file 2941861.f1.docx]

**Supplementary Table 1.** Quality assessment of the cross-sectional studies

| Author | Public Year | 1 | 2 | 3 | 4 | 5 | 6 | 7 | 8 | 9 | 10 | 11 |
| --- | --- | --- | --- | --- | --- | --- | --- | --- | --- | --- | --- | --- |
| Tanhauserováa | 2012 | + | - | - | - | - | + | + | + | + | + | - |
| Krzyzanowska | 2011 | + | + | + | - | 0 | + | + | + | - | + | - |
| Yonem | 2009 | + | + | + | + | - | + | + | + | - | - | - |
| Makino | 2008 | + | - | + | + | - | + | 0 | + | - | + | - |
| Krzyzanowska | 2007 | + | - | + | - | + | - | + | + | + | - | - |

Abbreviation: +: YES; -: NO; 0: not clear.1) Define the source of information (survey, record review);2) List inclusion and exclusion criteria for exposed and unexposed subjects (cases and controls) or refer to previous publications;3) Indicate time period used for identifying patients;4) Indicate whether or not subjects were consecutive if not population-based;5) Indicate if evaluators of subjective components of study were masked to other aspects of the status of the participants;6) Describe any assessments undertaken for quality assurance purposes (e.g., test/retest of primary outcome measurements);7) Explain any patient exclusions from analysis;8) Describe how confounding was assessed and/or controlled;9) If applicable, explain how missing data were handled in the analysis;10) Summarize patient response rates and completeness of data collection;11) Clarify what follow-up, if any, was expected and the percentage of patients for which incomplete data or follow-up was obtained.

**Supplementary Table 2.** Quality assessment of the case-control studies

| **First author** | **Representativeness of the cases** | **Case definition adequate** | **Ascertainment of exposure** | **Same method of ascertainment for cases and controls** | **Control for important factor or additional factor** | **Selection of Controls** | **Definition of Controls** | **Non-Response rate** | **Total quality scores** |
| --- | --- | --- | --- | --- | --- | --- | --- | --- | --- |
| Jayachandran | ☆ | ☆ | ☆ | ☆ | -- | ☆ | ☆ | ☆ | 7 |
| Bekpinar | ☆ | ☆ | ☆ | ☆ | -- | ☆ | ☆ | ☆ | 7 |
| Yasar | ☆ | ☆ | ☆ | ☆ | -- | ☆ | ☆ | ☆ | 7 |
| Abhary | ☆ | ☆ | ☆ | ☆ | -- | ☆ | ☆ | ☆ | 7 |
| Malecki | ☆ | -- | ☆ | ☆ | -- | ☆ | -- | ☆ | 5 |
